# Assessing Statistical Significance in Microarray Experiments Using the Distance Between Microarrays

**DOI:** 10.1371/journal.pone.0005838

**Published:** 2009-06-16

**Authors:** Douglas Hayden, Peter Lazar, David Schoenfeld, for The Inflammation and the Host Response to Injury Investigators

**Affiliations:** 1 Biostatistics Center, Massachusetts General Hospital, Boston, Massachusetts, United States of America; 2 Harvard School of Public Health, Boston, Massachusetts, United States of America; University of Michigan, United States of America

## Abstract

We propose permutation tests based on the pairwise distances between microarrays to compare location, variability, or equivalence of gene expression between two populations. For these tests the entire microarray or some pre-specified subset of genes is the unit of analysis. The pairwise distances only have to be computed once so the procedure is not computationally intensive despite the high dimensionality of the data. An R software package, permtest, implementing the method is freely available from the Comprehensive R Archive Network at http://cran.r-project.org.

## Introduction

We propose permutation tests based on the matrix of pairwise distances between microarrays to compare location, variability, or equivalence of gene expression between two populations. These tests can be applied to the entire genome or any subset of genes of interest. Thus they can be used as global tests of difference in gene expression or as a testing method applicable to gene set analysis.

These tests have several advantages over permutation tests based directly on the gene expression data. First, they are not computationally intensive because they reduce the high dimensional expression data to the low dimensional distance matrix and this only has to be done once. Second, this same reduction in dimensionality results in a reduction in the dimensionality of the potential nuisance parameters. Thus, the assumption of exchangeability between groups, which ensures the validity of the permutation test, only has to apply to the pairwise distances not to the entire microarray.

Many gene expression studies are designed to detect differential gene expression in two clinical or biological populations. Interest may focus on individual genes, specified groups of genes, or all genes sampled by a particular microarray. Early efforts were devoted to individual gene analysis [1]. A variety of test statistics were proposed and an eventual consensus emerged on the use of false discovery rate estimation to correctly account for multiple testing [2].

In order to incorporate existing biological information into the analysis of differential gene expression more recent work has been devoted to methods that treat sets of related genes as the unit of analysis [3]. Many methods and tools have been developed to analyze sets of genes [4], [1], [5]. These include methods which base inference on lists of genes which individually exceed a specified cut off threshold for significance and those which base inference on scores which combine information over the entire gene set [5]. Further, gene set methods can be divided into competitive and self-contained tests [3]. Competitive tests compare the specified gene set to the remaining genes outside the set while self-contained tests depend only on the specified genes. Competitive tests have been shown to be conceptually flawed [3] and will not be considered further.

A number of self-contained, multivariate, tests have been proposed [6], [7], [8]. Any multivariate test requires a test statistic which aggregates information over all genes in the gene set and a method of inference to determine if the observed magnitude of the test statistic is extreme under the null hypothesis of no between group difference in gene expression. The primary challenge in developing these multivariate tests is posed by having relatively few samples and many genes. In particular, asymptotic methods may not be applicable and the sample gene covariance matrix may not be full rank and is therefore not invertible.

Kong *et al.* [7] discuss several modifications to Hotelling's T^2^ [9] and suggest reducing the gene set to the lower dimensional subspace of principal components in which the sample gene covariance matrix becomes invertible. A permutation test is used for inference.

Goeman *et al.* [6] circumvent the problem of inverting the sample gene covariance matrix by developing a test statistic derived from a score test for a logistic regression model. In this model the gene expression values are considered to be fixed constants and the group indicator variable is considered to be stochastic. Their statistic is proportional to Hotelling's T^2^ under the assumption of equal variance and zero covariance across all genes. They present the statistic's asymptotic distribution but suggest a permutation test for inference in small samples.

Hummel *et al.* [8] propose a test statistic which combines residual sums of squares from gene-wise linear regression models, which may include additional covariates of interest, fit with and without the group effect. They present the asymptotic distribution of the test statistic under the assumption that the genes follow a multivariate normal distribution and use a shrinkage estimator to regularize the sample gene covariance matrix. They also propose a permutation test based on the residual gene expression derived by regressing out an intercept and the additional covariates gene by gene. In the absence of additional covariates, the resulting statistic can be shown to be permutationally equivalent to Hotelling's T^2^ under the assumption of equal variance and zero covariance across all genes. Note that two statistics are permutationally equivalent [10] if they have the same (or reverse) ordering over all permutations and therefore always give the same permutation p-value.

In this paper we propose methods to test for a significant difference in gene expression between two study groups based on the matrix of pairwise distances between microarrays. In the following sections we present the test statistics given the assumption of Euclidean distance, additive errors, and a completely randomized design. We also present an application to respiratory recovery in trauma patients and extensions to paired and blocked designs as well as the use of a distance measure based on correlation.

## Methods

### Introduction

Consider an experiment comparing gene expression in two populations. The gene expression values, or some function of the gene expression values which measures the biological signal for each gene, can be represented by two groups of column vectors. We will represent these column vectors by 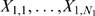 and 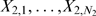, for groups 1 and 2 respectively where 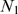 and 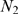 are the number of arrays in groups 1 and 2 respectively. For simplicity we will refer to these vectors as microarrays. Let 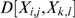 be the dissimilarity or distance between two microarrays.

Inference concerning the location and variability of groups 1 and 2 can be based on the three means: 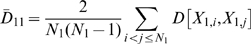
(1)

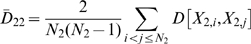
(2)

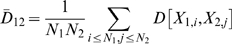
(3)Where

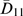
 is the mean distance between microarrays within group 1.

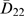
 is the mean distance between microarrays within group 2.

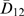
 is the mean distance between microarrays between groups 1 and 2.

### Additive Errors and Euclidean Distance

In this section we derive the expected value of 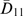, 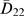, and 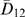 assuming a completely randomized design, an additive error model, and squared Euclidean distance. Under these assumptions we have: 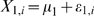
(4)

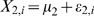
(5)where *μ*_1_ and *μ*_2_ are the respective mean vectors of the microarrays in groups 1 and 2 and *ε_1,i_* and *ε_2,i_* are random error vectors with expected value 0 and variance covariance matrices Σ_1_ and Σ_2_ respectively. The errors are assumed to be independent across microarrays.

The squared Euclidean distance between any two microarrays, *X_i,j_* and *X_k,l_* is 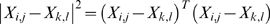
(6)and a simple calculation yields 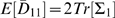
(7)

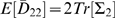
(8)

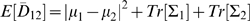
(9)where 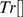 is the trace operator which gives the sum of the diagonal elements of a matrix.

We can now define a test statistic to compare the location or variability of groups 1 and 2.

To compare location let: 
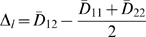
(10)


To compare variability let: 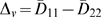
(11)These test statistics have expected values: 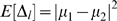
(12)and: 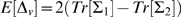
(13)


Inference concerning the magnitude of Δ*_v_* and Δ*_l_* can be made using a permutation test. Each permutation consists of assigning 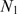 microarrays to group 1 and the remaining 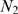 to group 2. Note that for each permutation the pairwise distances are simply re-indexed, they do not have to be recalculated. Only the values of 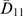, 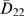, and 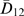 and Δ*_v_* and Δ*_l_* have to be recalculated based on the re-indexing.

Let 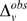 and 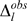 be the observed values of Δ*_v_* and Δ*_l_* and let 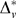 and 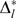 be the values from a permutation. If there are a total of B permutations, and assuming 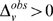 then 
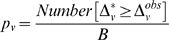
(14)is a one-sided p-value [11] for rejecting the null hypothesis that 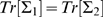. If 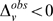 then the inequality in Equation 14 is simply reversed. Similarly 
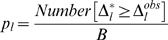
(15)is a one-sided p-value for rejecting the null hypothesis that 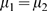.

Sometimes investigators design an experiment to compare a new technical method, such as sample preparation or target hybridization, to a proven “gold standard”. In such an experiment interest centers on showing that the new method is equivalent to the gold standard. To be equivalent it should not differ in mean and not exhibit greater variability. Assuming that the microarrays in group 1 were prepared using the gold standard, a summary statistic which can be used to reject the null hypothesis of equivalence is given by: 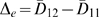
(16)If this statistic is large, then group 2 either has a different mean or more variability than group 1. This can easily be seen from its expected value under mean squared Euclidean distance: 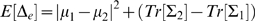
(17)Inference concerning the magnitude of Δ*_e_* can also be made using a permutation test. It should be noted that the statistics Δ*_v_*, Δ*_l_*, and Δ*_e_* are all special cases of Mantel's U statistic [12], for which he derives the permutation variance, and Δ*_l_* is similar to a special case of the MRPP statistic [13].

### Paired Design

In this section we consider modifications to the test statistics and permutation test to account for pairing in the experimental design. We still assume an additive error model and squared Euclidean distance.

Suppose the experimental design is paired so that each microarray in group 1 is paired with a microarray in group 2. In this case 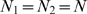 and we assume that 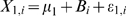
(18)

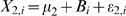
(19)where *B_i_* is a random vector, independent of all other quantities, with expected value 0 and variance covariance matrix Σ*_B_*. The quantities *μ*_1_, *μ_2_*, *ε_1,i_*, and *ε_2,i_* are again defined as in Section.

To account for the pairing we drop the within pair distance in our test statistics since it will be systematically less than the between pair distances. Thus we re-define 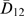 as: 
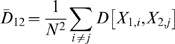
(20)then: 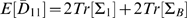
(21)

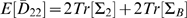
(22)

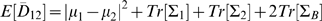
(23)so that Δ*_v_* and Δ*_l_* still have the same expected values given in Equations 13 and 12.

For this paired design, only paired microarrays are exchangeable and the permutation test must respect this structure. Thus, a permutation consists of exchanging microarrays across groups 1 and 2 within each pair of an arbitrary subset of paired microarrays.

### Blocked Design

In this section we consider modifications to the test statistics and permutation test to account for blocking in the experimental design. We still assume an additive error model and squared Euclidean distance.

Suppose the experiment consists of K blocks of related microarrays. In this case 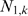 and 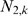 are the number of microarrays in block k in group 1 and 2 respectively. We also assume that for blocks 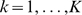


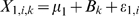
(24)

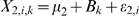
(25)where *B_k_* is a random vector , independent of all other quantities, with expected value 0 and variance covariance matrix Σ*_B_*. The quantities *μ*_1_, *μ*_1_, *ε_1,i_*, and *ε_2,i_* are again defined as in Section.

For this blocked design we need to modify the mean distances 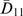, 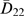, and 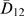 so that they depend only on within block distances. That is: 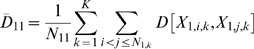
(26)

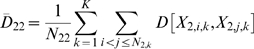
(27)

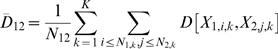
(28)where: 
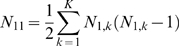
(29)

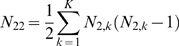
(30)

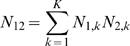
(31)


The permutation test for this design must respect the blocked structure. Microarrays are exchanged across groups only within each block.

### Negative Log Correlation Distance

There are various possible measures of distance between microarrays besides squared Euclidean distance. In this section we consider a measure based on the Pearson product moment correlation [14] which we will denote by 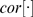. Assuming an additive error model and a completely randomized design, the correlation between two microarrays, 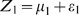 and 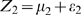, is 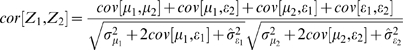
(32)where 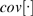 is the sample covariance, σ^2^ is the variance, and 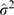 is the sample variance. By dividing the numerator and denominator in Equation 32 by 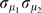 and rearranging some terms we get: 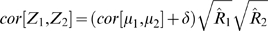
(33)where 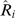 is the sample estimate of the reliability [15]

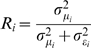
(34)and 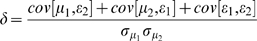
(35)with *E*[*δ*] = 0.

If the correlation is positive and under the assumption that *E*[*δ^2^*] is negligibly small then, if we define the distance between Z_1_ and Z_2_ by 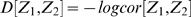
(36)then, the expected value of D[Z_1_, Z_2_] is approximated by 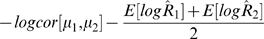
(37)


Defining Δ*_v_* and Δ*_l_* as above in Equations 11 and 10, these test statistics have expected values 
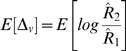
(38)and 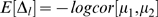
(39)so that Δ*_v_* estimates the log of the between group reliability ratio and Δ*_l_* estimates the negative log of the correlation between the group means.

Using the negative log correlation distance the three designs considered above need no modification except in the interpretation of the statistics Δ*_v_* and Δ*_l_* as presented in Equations 38 and 39. In the paired design it is convenient to think of the random pair effect, *B_i_* , as being absorbed in the error term. In the blocked design it is convenient to think of the random block effect, *B_k_* , as being absorbed in the mean.

Note that the reliability given by Equation 34, which can be interpreted as a signal to noise ratio, is a reasonable measure of the within group distance or variability, but it depends on both the mean, *μ*, and the error, *ε*. Thus a significant difference in the within group distance as measured by Δ*_v_* can be due to a difference in either the mean or the error.

A commonly used distance measure is one minus the correlation between microarrays. This distance measure is nearly equivalent to the negative log correlation distance for values of the correlation near one. However, by using this distance, the corrected distance between groups, Δ*_l_*, does not have the correct expected value and does not estimate the error free distance between the groups.

### Permutation Test Assumptions

Permutation tests may place weaker distributional assumptions on the data than do parametric tests but they are not totally free of assumptions. In general they only test the global null hypothesis, 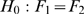 where *F*_1_ is the data generating distribution for group 1 and *F*_2_ is the data generating distribution for group 2. In particular they cannot test that a specific parameter of the data generating distribution differs between two groups without highly restrictive assumptions about the remaining (nuisance) parameters.

They do have the desirable property of being exact tests provided that, under the null hypothesis, the observations are exchangeable so that the joint distribution of the combined data set is invariant under permutations of the observation labels. The permutation tests we propose first reduce the extremely high dimensionality of the data (and attendant high dimensionality of the nuisance parameters) to the low dimensional matrix of pairwise distances. Since inference is based solely on the distribution of these distances the global null hypothesis becomes the much less restrictive assumption that the pairwise distances are identically distributed. Thus, only pairwise distances need be exchangeable by permutation of observation labels.

In particular, under Euclidean distance and the additive error model proposed above, the location permutation test, which tests the null hypothesis, 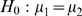, is exact under the relatively weak assumption that the inner product of any two distinct error terms is exchangeable. That is, the test is exact if terms of the form 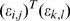, with 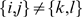, are exchangeable across observations. These scalar terms are easily shown to be simply random errors in the sense that they each have expected value zero and are uncorrelated with all other terms.

## Results

As an application of our proposed method we analyzed time to respiratory recovery in ventilated trauma patients in a data set previously described by Rajicic [16]. Patients were followed for 28 days post trauma and Affymetrix U133+2 microarrays were prepared from whole white blood cells sampled at days 0, 1, 4, 7, 14, 21, 28. We considered a subset of 48 ventilated patients who had a day one sample and divided them into two subgroups: those who recovered from ventilation prior to day seven (early recovery, N = 22) and those who did not (late recovery, N = 26). Of clinical interest is the potential association of inflammation on day one and subsequent respiratory recovery. To address this issue, a set of 445 probesets whose GO annotation included the term “inflammatory” was retrieved for analysis by a keyword search of the Affymetrix web site (http://www.affymetrix.com/index.affx).

**Figure 1:**
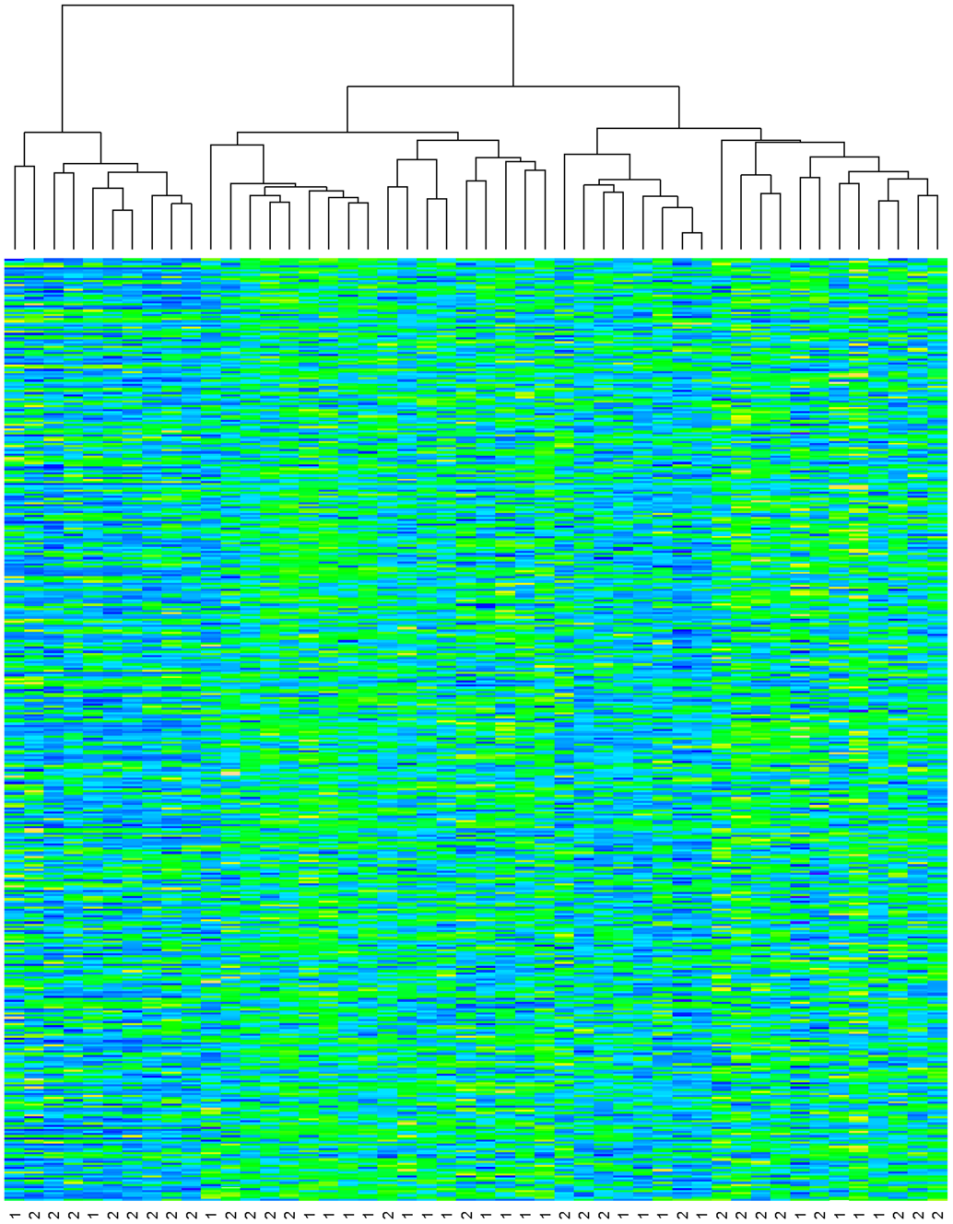
Heat Map

Figure 1 shows a heat map of day one gene expression for the 48 patients (columns) over the 445 probesets (rows). In the figure, columns labeled with a “1” comprise the early recovery group, those labeled with a “2” comprise the late recovery group. Hierarchical clustering using Euclidean distance has been applied to the patients but not the probesets.

As can be seen in the figure, it is not readily apparent that the clustering separated the early and late recovery groups. However, the highest split separated the patients into one group (right hand side of figure) having 20/38 (53%) early recovery patients while the remaining group (left hand side off figure) had only 2/10 (20%) early recovery patients. This imbalance may be suggestive of a difference in gene expression between the early and late recovery groups.

To formally test for a group effect we applied our proposed permutation test for a Euclidean distance location difference between the early and late recovery groups and obtained a one sided p-value of 0.0168, indicating a significant difference in gene expression.

As a check on this result, in the spirit of Kong's use of Hotelling's T-square in the principal component space [7], we applied MANOVA to the first three principal components. We obtained a two sided p-value of 0.0393 for the group effect, a result similar to the permutation test result. The choice of three principal components was arbitrary, however, and results vary with the number chosen for analysis.

**Figure 2:**
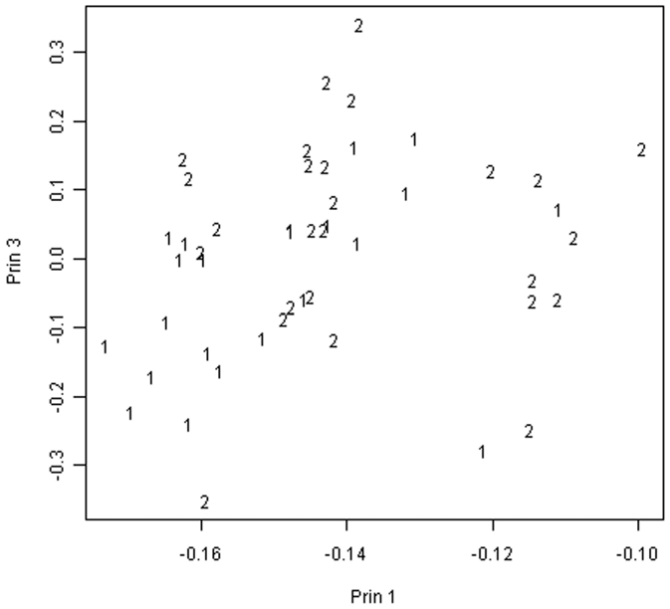
Principal Components

Figure 2 illustrates the location of the two recovery groups in the subspace spanned by the first and third principal components. Note that the same cluster of two early recovery patients (denoted by “1”) and eight late recovery patients (denoted by “2”) can be seen on the right hand side of the figure.

Similar results were obtained using the globaltest R-package of Goeman *et al.* [6], with a p-value of 0.0167 as well as the GlobalAncova R-package of Hummel *et al.* [8], with a p-value of 0.0210.

Finally it may be of interest to note that, for the remaining 54230 probesets not associated with inflammation, the group difference was marginally significant with a p-value of 0.0462 by our method and p-values of 0.0508 and 0.0480 by the methods of Goeman and Hummel respectively. This suggests that factors other than inflammation may also be associated with time to ventilator recovery.

## Discussion

Our proposed test statistics attempt to divide differences in gene expression into differences in location and differences in variation. The hope is to find tests for one parameter when the other parameter differs. This is a common statistical problem that only has a solution under limited circumstances. For instance, if data are normally distributed it is possible to find an exact test for variability differences when there are differences in location but testing for differences in location when there are differences in variability is the famous Behrens-Fisher problem which has no optimal small sample solution [17]. We use permutation tests because the distribution functions of gene expression are unknown. The permutation tests we propose have the advantage that the pairwise distances are calculated before the permutations are applied so the tests are not computer intensive. The location test is designed to have power for a location difference and correct Type I error rate even given a variability difference, and the variability test is designed to have the opposite characteristics. Simulations (results not shown) show that, in the cases that we simulated, we succeeded. However, there may be circumstances where variability differences appear as location differences and vice-versa.

Since the distribution functions of gene expression are unknown it is not possible to calculate the power of the location test under specified alternative hypotheses. However, as pointed out in the Introduction, for a two group comparison, both Goeman's and Hummel's methods are permutationally equivalent to Hotelling's T^2^ under the assumption of equal variance and zero covariance across all genes. This holds true for our location test as well, using Euclidean distance in a balanced completely randomized design. Since permutational equivalence holds regardless of the distribution of the data, in the case of balanced completely randomized designs all three tests have equal power as permutation tests. In the moderately unbalanced case, with similar within group variability, simulations (not shown) show that our test is still nearly permutationally equivalent to the other two tests so that the power will be similar.

Our proposed location test is based on the matrix of pairwise distances between microarrays and is therefore related in a natural way to cluster analysis which applies an algorithm to the distance matrix to find clusters in the data. In experimental designs involving two predetermined groups of microarrays cluster analysis can be used as a graphical technique to see if the clustering algorithm “finds” the predetermined groups. Our proposed method can be thought of as a formal significance test of whether two predetermined groups form two distinct clusters. Thus our test is consistent with an intuitive visual display of the data. Of course applying our location test, or any significance test, to two groups that were discovered using a clustering algorithm would be circular and therefore invalid.
